# The RNA Demethylase ALKBH5 Maintains Endoplasmic Reticulum Homeostasis by Regulating UPR, Autophagy, and Mitochondrial Function

**DOI:** 10.3390/cells12091283

**Published:** 2023-04-29

**Authors:** Panneerdoss Subbarayalu, Pooja Yadav, Santosh Timilsina, Daisy Medina, Kunal Baxi, Robert Hromas, Ratna K. Vadlamudi, Yidong Chen, Patrick Sung, Manjeet K. Rao

**Affiliations:** 1 Greehey Children’s Cancer Research Institute, UT Health San Antonio, San Antonio, TX 78229, USA; 2 Department of Cell Systems and Anatomy, UT Health San Antonio, San Antonio, TX 78229, USA; 3 Department of Medicine, UT Health San Antonio, San Antonio, TX 78229, USA; 4 Department of Obstetrics and Gynecology, UT Health San Antonio, San Antonio, TX 78229, USA; 5 Department of Biochemistry and Structural Biology, UT Health San Antonio, San Antonio, TX 78229, USA

**Keywords:** ALKBH5, m6A, unfolded protein response, mitochondria, ERLIN1, ER homeostasis, autophagy

## Abstract

Eukaryotic cells maintain cellular fitness by employing well-coordinated and evolutionarily conserved processes that negotiate stress induced by internal or external environments. These processes include the unfolded protein response, autophagy, endoplasmic reticulum-associated degradation (ERAD) of unfolded proteins and altered mitochondrial functions that together constitute the ER stress response. Here, we show that the RNA demethylase ALKBH5 regulates the crosstalk among these processes to maintain normal ER function. We demonstrate that ALKBH5 regulates ER homeostasis by controlling the expression of ER lipid raft associated 1 (ERLIN1), which binds to the activated inositol 1, 4, 5,-triphosphate receptor and facilitates its degradation via ERAD to maintain the calcium flux between the ER and mitochondria. Using functional studies and electron microscopy, we show that ALKBH5-ERLIN-IP3R-dependent calcium signaling modulates the activity of AMP kinase, and consequently, mitochondrial biogenesis. Thus, these findings reveal that ALKBH5 serves an important role in maintaining ER homeostasis and cellular fitness.

## 1. Introduction

M^6^A (*N*^6^-methyladenosine) RNA methylation is one of the most predominant modifications observed in mammalian mRNA. M^6^A is dynamically modulated by the multi-component RNA methyltransferase complex, which methyltransferase-like 3 (METTL3), methyltransferase-like 14 (METTL14), and WT1 associated protein (WTAP); the RNA demethylases alkb homolog 5 (ALKBH5) and fat mass obesity-associated (FTO); m^6^A readers, such as YTH N6 methyladenosine RNA binding protein (YTHDF), and the insulin-like growth factor 2 mRNA binding protein (IGF2BP) family of proteins. Prior studies have shown that m^6^A may affect RNA stability, metabolism, and splicing [1,2,3]. M^6^A is involved in various biological processes, including transcription/translation, stem cell self-renewal [4], cell fate transition [5], and circadian rhythm [6]. M^6^A methylation is also reported to play equally important roles in tumorigenesis [7,8]. In particular, we and others have shown that m^6^A writers (RNA methyltransferases) and erasers (RNA demethylases) support the growth and proliferation of tumors from diverse lineages [7,8]. These observations indicate that cancer cells may be dependent upon RNA methylation machinery for their survival. Despite these advances, much need still remains to understand how m^6^A may help cancer cells to cope with the challenges they must overcome to survive and thrive. Even less is known about the role of specific m^6^A writers and erasers in regulating cell survival mechanisms. In this study, we report that RNA demethylase ALKBH5 regulates endoplasmic reticulum (ER) homeostasis and adaptive survival mechanism by controlling the unfolded protein response (UPR), autophagy, and mitochondrial function in normal and cancer cells.

Normal and cancer cells have devised well-orchestrated adaptive mechanisms such as the UPR to endure environmental or oncogenic stresses. The UPR is a pro-survival mechanism triggered by the accumulation of unfolded or misfolded proteins in the ER lumen. The UPR is induced by the ER-anchored transmembrane sensor proteins inositol-requiring enzyme 1 (IRE1), protein kinase R (PKR)-like endoplasmic reticulum kinase (PERK) and activating transcription factor 6 (ATF6). The activated UPR sensors upregulate protein folding machinery by activating their transducers X-box-binding protein 1 (XBP1) (for IRE1), activating transcription factor 4 (ATF4) (for PERK), and cleaved ATF6 (ATF6). Activation of the UPR induces degradation of misfolded proteins using the ubiquitin-proteosome system (ERAD) or autophagy-dependent degradation pathways to alleviate ER stress and restore ER homeostasis. A direct link between the UPR and autophagy is supported by findings that the UPR transducer ATF4 transactivates several genes associated with autophagy. Furthermore, the UPR-associated protein PERK is critical for autophagy induction following ER stress. Along with the induction of autophagy, altered mitochondrial function is inextricably linked with ER stress and the UPR. For example, ATF4 and ATF6 regulate mitochondrial function and biogenesis [9,10,11]. Moreover, mitochondria-associated membranes (MAMs), which are areas of close contact between the ER and mitochondria, provide an elaborate platform for ER signaling, including the maintenance of Ca^2+^ flux between the mitochondria and ER. Furthermore, MAMs are important in regulating autophagy because they are a source of the autophagosomal membrane [12]. These observations suggest that crosstalk among the UPR, autophagy, and mitochondria is critical for maintaining ER homeostasis. Despite significant progress made in understanding each of these discrete events, how this crosstalk among different stress responses may regulate ER-associated signaling is poorly understood. In this study, we report that RNA methylation may be one of the important regulators of global ER-associated stress responses in both normal and cancer cells.

## 2. Materials and Methods

Cell culture: The human cancer cell lines 143B, MG63, U2OS, HEK293T, HeLa, MDA-MB-231, and MDA-MB-468 were all purchased from the American Type Culture Collection (ATCC) and cultured in the standard growth medium, according to their guidelines. IMR-90 cells (lung fibroblasts) were kindly provided by Dr. Gregory Aune’s laboratory (UT Health San Antonio). All cell lines were maintained in a humidified incubator at 37 °C and 5% CO_2._

RNA and protein: Total RNA extracted from cell lines was subjected to qRT-PCR. Protein samples for Western blots were prepared from total cell lysates. Cancer cell lines were transfected with scrambled-siRNA or ALKBH5-siRNA (Sigma-Aldrich, St. Louis, MO, USA) for 48 or 72 h before they were subjected to qRT-PCR or Western blot analysis, as described previously [7]. The antibodies, plasmids, and reagents used in this study are listed in Table S1.

RNA sequencing: RNA sequencing on total RNA from scrambled-siRNA- and ALKBH5-siRNA-transfected 143B cells was carried out according to the manufacturer’s protocol (Illumina Inc., San Diego, California, USA) and as we previously described [7]. Upregulated and downregulated genes were determined by the following criteria: (i) absolute log_2_ fold change > 1, [13] average reads per kilobase per million mapped reads > 1, and (ii) multiple-test adjusted (Benjamini–Hochberg) *p*-value < 0.05. Raw data were deposited in the Gene Expression Omnibus database (GSE154530).

Methyl RNA immunoprecipitation sequencing (Me-RIP-seq): Me-RIP-seq on mRNA isolated from scrambled and ALKBH5-siRNA transfected 143B cells was performed, as we described previously [7]. Briefly, total RNA was isolated from 143B cells transfected with either scrambled or siALKBH5 for 48 h, using TRIzol reagent (Invitrogen, Waltham, MA, USA, Cat # 15596-026). Total RNA was used to isolate mRNA using Dynabeads™ mRNA DIRECT™ Purification Kit (Invitrogen Cat # 61012). Isolated mRNA was quantified and 2 µg mRNA was fragmented to 100–200 bp in length using a Bioruptor (Diagenode, Denville, NJ, USA,). A portion of mRNA was saved for the input sample. For m^6^A RNA immunoprecipitation, fragmented mRNA was incubated in immunoprecipitation buffer with rabbit anti-m^6^A antibody (Synaptic Systems, Goettingen, Germany, Cat #202003) for 2 h in the cold room. Enriched M^6^A mRNA was used for RNA sequencing.

Me-RIP-seq was performed on immunoprecipitates generated using three different anti-m^6^A antibodies (Synaptic Systems, Goettingen, Germany, Cat #202003; MABE1006, and ABE572 (both, Millipore-Sigma, Burlington, MA, USA)).

MeRIP-seq and data preprocessing: Equal amounts of barcoded samples were submitted for cluster generation on an Illumina cBot Cluster Station before sequencing on an Illumina HiSeq 3000 system using the 50SR sequencing module, according to the manufacturer’s instructions and as we described previously [7]. We applied the MeTPeak program [14] to identify m^6^A enrichment sites and used Guitar tools [15,16] to generate peak distributions. We used the MEME-ChIP program [17] for m^6^A motif enrichment analyses. Differential m^6^A methylation analyses from MeRIP-seq data were carried out using methods similar to those in our earlier publications [7,18]. We used our novel algorithm MeTDiff [14] for methylation peak calling and differential peak identification. MeTDiff identifies methylation fold-change of the percentages of methylated fragments for a specific mRNA locus along with their statistical significance (*p*-values) and multiple-test adjusted *p*-values. A positive value indicates high (hyper-) m^6^A methylation in the treated versus untreated condition, whereas a negative value indicates lower (hypo-) methylation.

RNA-binding protein immunoprecipitation: RNA-binding protein immunoprecipitation to show ALKBH5 interaction with ERLIN1 transcripts was carried out using a Magna RIP™ RNA-Binding Protein Immunoprecipitation Kit (Sigma, USA, Cat#17-700), according to the manufacturer’s protocol.

GFP-LC3B-RFP Autophagy Flux. To understand the role of ALKBH5 in autophagy flux, cells were transfected with RFP-LC3B-GFP plasmid for 36–48 h in ALKBH5-depleted cells. Cells were fixed, mounted with DAPI, and observed under an epifluorescence microscope [19]. RFP-GFP-LC3 fluorescent-tagged LC3 is routinely used to study the autophagic flux. RFP-GFP-LC3 emits both green and red fluorescence signals when the protein localizes to autophagosomes. An increase in red puncta reflects autophagy induction, while an increase in yellow puncta reflects inhibition of autophagosome maturation and/or fusion with the lysosome [13].

Immunofluorescence: For immunofluorescence analyses, cells were fixed in 4% paraformaldehyde (Santa Cruz Biotechnology, Dallas, TX, USA) for 15 min at room temperature. After fixation, cells were blocked and permeabilized in 0.1% TritonX-100 in 5% normal goat serum/PBS for 30 min. After blocking and permeabilization, cells were incubated with primary antibody diluted to 1:200 with 5% NGS in PBS and left overnight at 4 °C. Then, the cells were washed, and a secondary antibody conjugate was added with FITC/TRITC (1:500 dilution) for 1 h at room temperature. Cells were washed with PBS, three times for 5 min, and mounted with DAPI in ProLong Gold Anti-fade (Thermo Fisher Scientific, Waltham, MA, USA, Cat# P36930). Samples were analyzed with fluorescence and confocal microscopy (LSM 780 microscope), equipped with a 60× oil immersion 1.40 numerical aperture objective using Zen software (Carl Zeiss Ltd., Cambridge, UK).

Electron microscopy: For transmission electron microscopy, cells were transfected with scrambled or ALKBH5-siRNA for 72 h, fixed with glutaraldehyde, and processed at the UT Health San Antonio Electron Microscopy core facility.

Statistical analyses: All values and error bars in graphs represent means ± SEM. Respective *n* values are indicated in the figure legends. The *p*-values were determined by two-tailed Student’s *t* tests and analysis of variance, as required for appropriate statistical testing.

## 3. Results

### 3.1. ALKBH5 Regulates the Unfolded Protein Response (UPR) and Autophagy

**Figure 1:**
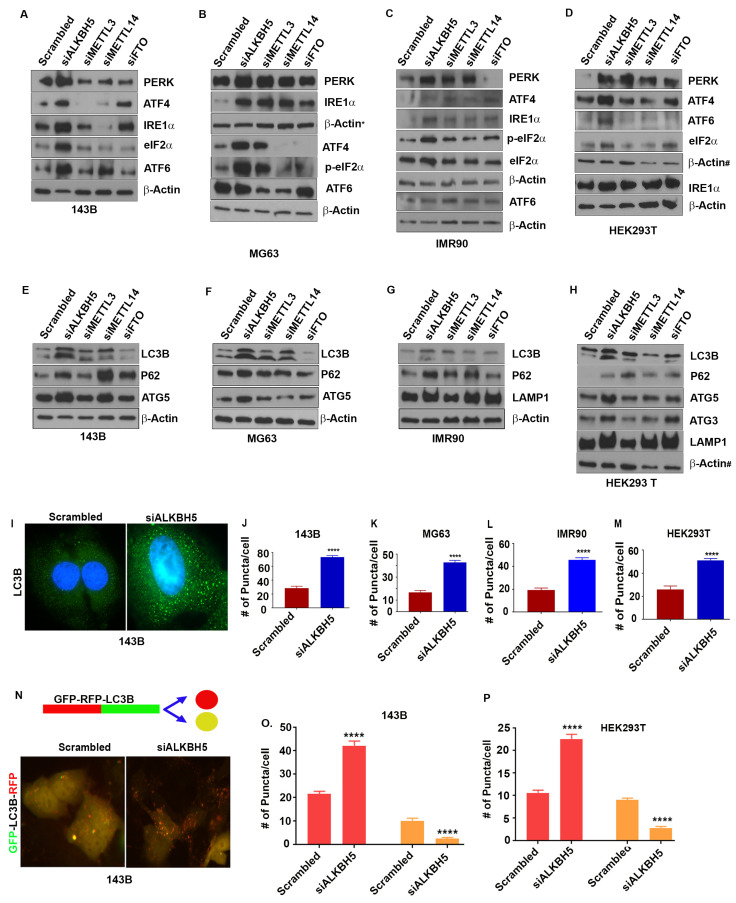
(**A**–**D**) Western blot analyses of 143B (**A**), MG63 (**B**), IMR90 (**C**), and HEK293T (**D**) cells transfected with scrambled-siRNA (scrambled) or ALKBH5-siRNA (siALKBH5), METTL3-siRNA (siMETTL3), METTL14-siRNA (siMETTL14), and FTO-siRNA (FTO) using antibodies against indicated proteins. β-actin was used as a loading control. (**E**–**H**) Western blot analyses of autophagy-related protein in 143B (**E**), MG63 (**F**), IMR90 (**G**), and HEK293 (**H**) transfected with scrambled, siALKBH5, siMETTL3, siMETTL15, or siFTO using antibodies against the indicated proteins. β-actin was used as a loading control. Gel photographs shown in (**A**–**H**) are representative of at least three independent experiments. (**I**) Immunofluorescence analysis of LC3 puncta in scrambled and siALKBH5 transfected 143B cells. (**J**–**M**) Numbers of puncta in 143B (**J**), MG63 (**K**), IMR90 (**L**), and HEK293T (**M**) cells. A total of 100 individual cells/group were counted. (**N**) Top, schematic shows RFP-GFP fluorescent-tagged LC3 construct used for studying autophagic flux. Bottom, immunofluorescence analyses of autophagy flux in 143B cotransfected with RFP-GFP-LC3 and scrambled (siControl), or siALKBH5. Increased number of red puncta shows induction of autophagy flux. (**O**,**P**) Bar graphs showing the number of red and yellow puncta from immunofluorescence analysis, as shown above (**N**) in 143B (**O**), and HEK293T (**P**) cells cotransfected with RFP-GFP-LC3 and si-Control or siALKBH5. A total of 100 individual cells/group were counted. * Symbol next to β-actin in (**B**) indicate the same loading control as in Figure 2D. # symbol next to β-actin in (**D**,**H**) indicate the same loading control. The same loading controls were used because gels were stripped and reprobed for different proteins. Relevant proteins are shown in different figures to maintain the flow of the results. **** *p* < 0.0001.

To understand the role of m^6^A in ER-associated stress responses, we silenced the expression of the RNA methyltransferase complex proteins, METTL3 and METTL14, and the RNA demethylases ALKBH5 and FTO using siRNAs, and determined the levels of the UPR and autophagy proteins. Depletion of ALKBH5 increased the levels of the UPR sensor proteins PERK, ATF4, ATF6, and IRE-α (Figure 1A–D) in normal and cancer cell lines. Unlike ALKBH5, silencing of METTL3, METTL14, or FTO did not have consistent effects on the levels of the UPR proteins in the cell lines tested (143B, IMR90, HEK293T, MDA-MB-231, MDA-MB-468, MG63, HeLa, and U2OS) (Figure 1A–D). Furthermore, ALKBH5 silencing resulted in increased levels of activated eukaryotic translation initiation factor 2 α (eIF2α), which functions as a regulator of global translation in response to stress (Figure 1A–D). These results were further validated in multiple siRNAs against ALKBH5 (Figure S1A,B), and stable expression of control shRNA or shALKBH5 (Figure S1C).

UPR sensor proteins are reported as essential for autophagy induction following ER stress [20]. To test this concept, we determined the levels of autophagy-related proteins in various cancer cell lines. Similar to the UPR, the depletion of ALKBH5 resulted in increased levels of the autophagy marker protein LC3B (Figure 1E–H and Figure S1E). In contrast, the knockdown of FTO led to reduced levels of LC3B in some cell lines, while depletion of METTL3 or METTL14 did not consistently affect the levels of LC3B (Figure 1E–H). In addition to LC3B, ALKBH5 silencing led to increased levels of the autophagy-related proteins ULK1, ATG3, ATG5, ATG12, ATG16-L1, P62, VSP34, and LAMP1 (Figure 1E–H and Figure S1A). This increase in ALKBH5-silenced cells is likely due to the induction of the UPR since PERK-eIF2α-induced activation of ATF4 upregulates the transcription of many autophagy genes [20,21].

To further substantiate the role of ALKBH5 in autophagy, we assessed autophagic flux by measuring the percentage of LC3-containing autophagosomes in ALKBH5-silenced cells. Immunofluorescence analysis showed more LC3 puncta in ALKBH5-depleted cells (Figure 1I–M). In addition, RFP-LC3-GFP-expressing ALKBH5-siRNA transfected cells revealed a significantly increased number of RFP-LC3 puncta, suggesting an increased autophagic flux in ALKBH5-depleted cells compared to control cells (Figure 1N–P). To address whether ALKBH5 demethylase activity is critical in regulating the UPR and autophagy events, we performed rescue experiments. Overexpression of ALKBH5 decreased the levels of the UPR and autophagy-associated proteins, while overexpression of ALKBH5 with catalytic domain mutation had no effect on the UPR or autophagy-associated proteins (Figure S1D).

### 3.2. ALKBH5 Regulates ERLIN1 Expression in m^6^A-Dependent Manner

Given that the UPR induces autophagy after ER stress and dysregulated autophagy may induce IRE1 activity, and consequently, the UPR, our results suggested that ALKBH5 may be a critical regulator of the crosstalk among the ER stress, UPR, and autophagy. To address whether the demethylase activity of ALKBH5 may be critical in regulating ER-associated stress responses, we performed methyl RNA immunoprecipitation followed by deep sequencing (me-RIP-seq) on ALKBH5-silenced cells. Since ALKBH5 is an RNA demethylase, we focused on genes with enriched m^6^A peaks (called hyper m^6^A) in ALKBH5-silenced cells that are directly regulated by ALKBH5-mediated m^6^A demethylation. Surprisingly, UPR-associated genes showed no significant differences in m^6^A levels/peaks in ALKBH5-silenced versus scrambled-siRNA transfected cells, suggesting that the induction of ER stress may be regulated by other ALKBH5 target genes, such as ER lipid raft associated 1 (ERLIN1).

**Figure 2:**
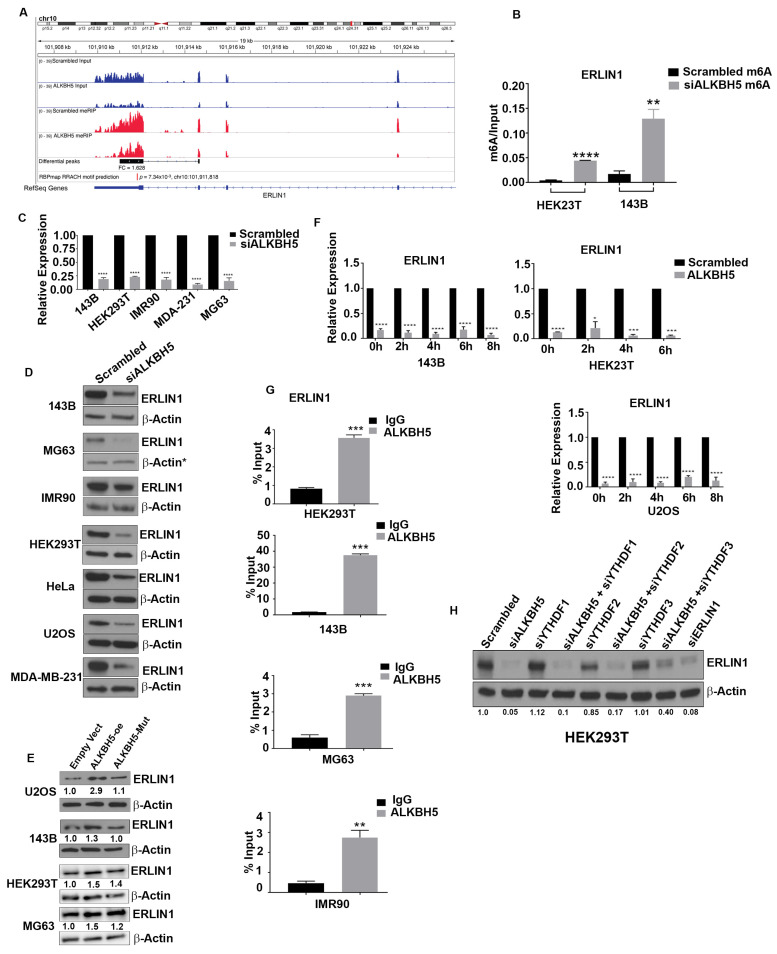
(**A**) MeRIP-seq analyses showing enriched ERLIN1 m^6^A peaks identified in scrambled-siRNA (SCR) and ALKBH5-siRNA (ALKBH5 KD)-transfected 143B cells. Blue tracks represent input and red tracks represent MeRIP for scrambled-siRNA and ALKBH5-siRNA transfected 143B cells. M^6^A fold-change values (differential peak track) were calculated by dividing the total m^6^A values with the expression levels of ERLIN1 for both scrambled and ALKBH5-siRNA transfected cells, using the MeTDiff program. ERLIN1 shows significantly increased m^6^A levels (adj *p*-value < 0.05) in ALKBH5-siRNA compared to scrambled-siRNA transfected 143B cells. M^6^A motif (shown as motif GGA C/A A/G) distribution was determined by subjecting Me-RIP-seq data to MEME-ChIP (E value = 2.2 × 10^−68^). (**B**) M^6^A RNA immunoprecipitation using an antibody against m^6^A followed by real-time PCR (Me-RIP-PCR) showed enrichment of ERLIN1 transcripts in siALKBH5 compared to scrambled-siRNA transfected cells. (**C**) QRT-PCR analysis of ERLIN1 expression, in indicated cell lines transfected with scrambled-siRNA or ALKBH5-siRNA, shown as mean ± SEM (*n* = 3 experiments). The *p*-values were calculated using standard Student *t*-tests. (**D**) Western blot analysis of ERLIN1 in scrambled-siRNA and ALKBH5-siRNA-transfected 143B, MG63, IMR90, HEK293T, HeLa, U2OS, and MDA-MB-231 cells using ERLIN1 antibody. β-actin was used as a loading control. (**E**) Western blot analysis of ERLIN1 in empty vector or ALKBH5 expression plasmid (with/without catalytic domain mutant) transfected 143B, HEK293T, and MG63 cells using ERLIN1 antibody. β-actin was the loading control. Gel photographs shown in D and E are representative of at least three independent experiments. (**F**) QRT-PCR analyses showing the stability of ERLIN1 mRNA in scrambled-siRNA or ALKBH5-siRNA transfected 143B, U20S, and HEK293T cells treated with actinomycin D (10 μg/mL) for the indicated hours. Transcript levels at 0 h were normalized to 1 for each time point. (**G**) RNA immunoprecipitation of total RNA isolated from HEK293T, 143B, MG63, and IMR90 cells using an antibody against ALKBH5. IgG served as a negative control. (**H**) Western blot analyses of HEK293 cells transfected with scrambled-siRNA, ALKBH5-siRNA, YTHDF1-siRNA, YTHDF2-siRNA, YTHDF3-siRNA, or co-transfected with ALKBH5-siRNA + YTHDF1/2/3-siRNA using an antibody against ERLIN1. For all panels, results are shown from analyses of at least three independent experiments.* Symbol next to β-actin in (**D**) indicate the same loading control as in Figure 1B. The same loading controls were used because gels were stripped and reprobed for different proteins. Relevant proteins are shown in different figures to maintain the flow of the results. **** *p* < 0.0001; *** *p* < 0.001; ** *p* < 0.01; * *p* < 0.05.

ERLIN1 is important in ER-associated signaling as it binds to activated inositol 1, 4, 5,-triphosphate receptors (IP3R) and marks them for degradation via ERAD to maintain calcium homeostasis. Moreover, impaired regulation of IP3R-dependent calcium flux is a key event associated with ER stress and UPR [22]. Methyl-RNA immunoprecipitation-seq results revealed that ERLIN1 was one of the top hyper-m^6^A genes in ALKBH5-silenced cells (Figure 2A). Consistent with that result, enriched m^6^A peaks overlapped with m^6^A binding motifs (RRACH) in the 3′ UTR of ERLIN1. These results were further validated in different cell lines by quantitative methyl-RNA immunoprecipitation PCR using different ALKBH5 siRNAs (Figure 2B). ALKBH5 silencing led to significantly reduced expression of ERLIN1 mRNA and protein in multiple cell lines (Figure 2C,D). To further verify the importance of ALKBH5 demethylase activity in regulating ERLIN1, we created an overexpressed ALKBH5 catalytic mutant construct and determined its effect on ERLIN1 expression. Unlike wildtype ALKBH5, the ALKBH5 mutant failed to elevate ERLIN1 levels (Figure 2E). However, not all cell lines showed robust effects on ERLIN1 levels in ALKBH5 mutant transfected cells when compared to the wildtype, suggesting that either the expression of the ALKBH5 catalytic mutant was not sufficient enough to offset the effect of the high levels of endogenous ALKBH5, or that ALKBH5 regulates the stability of ERLIN1 through a different mechanism in those cell lines.

Previous reports showed that hyper-m^6^A results in reduced stability of target mRNA transcripts. Since ALKBH5 KD increased m^6^A levels and significantly decreased ERLIN1 mRNA steady-state levels**,** we performed mRNA stability analyses in scrambled-siRNA and ALKBH5-silenced cells treated with actinomycin D (to inhibit de novo transcription). ALKBH5 silencing significantly reduced ERLIN1 mRNA stability compared to scrambled siRNA-treated cells (Figure 2F). Consistent with those findings, RNA immunoprecipitation showed that ALKBH5 binds to the ERLIN1 transcript (Figure 2G).

To further understand how m^6^A may regulate ERLIN1 expression, we sought to identify readers that either stabilized or degraded m^6^A-containing transcripts. The YTHDF family of proteins serves as readers of m^6^A by either stabilizing or degrading the target transcripts. To identify whether one or more YTHDF proteins may serve as a reader of the methylated ERLIN1 transcript, we performed rescue experiments. Silencing of ALKBH5 followed by silencing of either YTHDF1, 2, or 3 showed that only YTHDF3 rescued the effects of ALKBH5 knockdown on ERLIN1 expression (Figure 2H). Together, these results suggest that ALKBH5 silencing results in increased YTHDF3 availability, binding to methylated ERLIN1 mRNAs, and consequently, degradation.

### 3.3. ALKBH5 Regulates the ERLIN1-IP3R Signaling Axis to Regulate Calcium Flux and Mitochondrial Biogenesis

**Figure 3:**
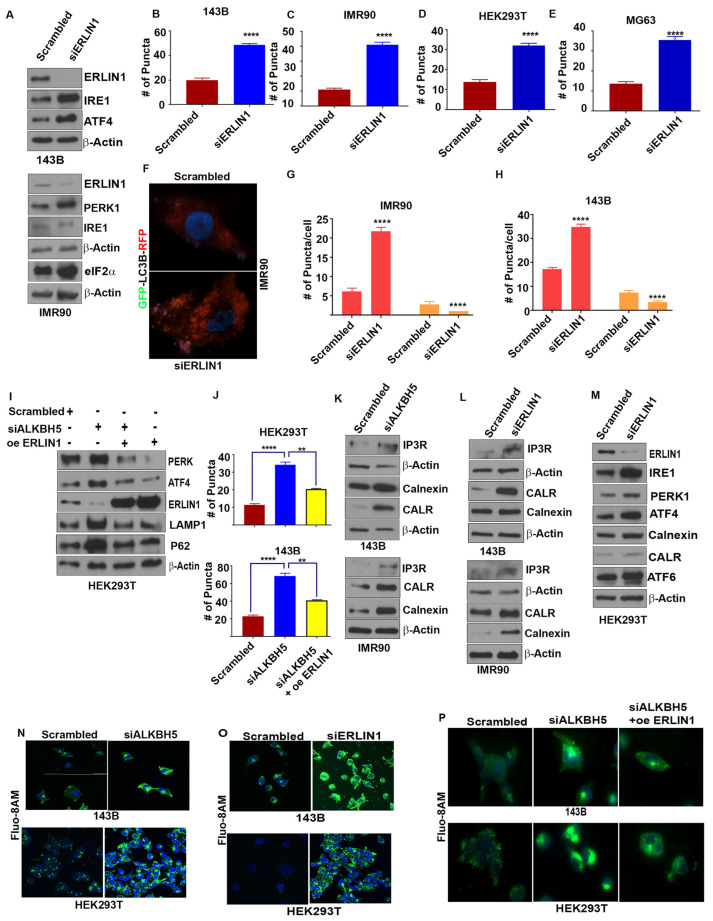
(**A**) Western blot analyses of UPR-associated genes in scrambled-siRNA (scrambled) or ERLIN-siRNA (siERLIN1) transfected 143B and IMR90 cells using antibodies against the indicated proteins. β-actin was used as the loading control. Gel photographs are representative of at least three independent experiments. (**B**–**E**) Average numbers of LC3 puncta/cell in 143B (**B**), IMR90 (**C**), HEK293T (**D**), and MG63 (**E**) cells transfected with siERLIN1. Puncta were counted in 100 cells/group. (**F**) Immunofluorescence analyses of autophagy flux in IMR90 cells cotransfected with RFP-GFP-LC3 and scrambled (siControl) or siERLIN1. (**G**,**H**) Bar graphs showing the number of red and yellow puncta from immunofluorescence analysis, as shown above (**F**) in IMR90 (**G**) and 143B (**H**) cells cotransfected with RFP-GFP-LC3 and siControl or siERLIN1. A total of 100 individual cells/group were counted. (**I**) Western blot analysis of 143B cells cotransfected with scrambled-siRNA (Scr), siALKBH5, or siALKBH5 + ERLIN1 overexpression plasmids (oe ERLIN1). Gel photographs are representative of at least three independent experiments. (**J**) Numbers of LC3 puncta in cells transfected with scrambled-siRNA, siALKBH5, or siALKBH5 + oe ERLIN1. (**K**–**M**) Western blot analyses of scrambled, siALKBH5 (**K**), or siERLIN1 (**L**,**M**) transfected 143B, IMR90, MG63, and HEK293T cells using antibodies against the indicated proteins. Gel photographs shown are representative of at least three independent experiments. (**N**,**O**) Confocal microscopic observation of intracellular calcium levels in scrambled-siRNA (siControl), siALKBH5, or siERLIN1 transfected 143B (**N**) and HEK293 cells (**O**). (**P**) Immunofluorescence analysis of intracellular calcium levels using Fluo-8AM in HEK-293 and 143B cells transfected with scrambled-siRNA (siControl), siALKBH5, or siALKBH5 + oe ERLIN1. (Calcium in Green color; nucleus stained with DAPI in blue color). Results are representative of at least three independent experiments. **** *p* < 0.0001; ** *p* < 0.01.

To elucidate how ALKBH5-ERLIN1 signaling may regulate the UPR, we first asked whether ERLIN1 may be directly involved in regulating the ER stress-mediated induction of the UPR. Silencing of ERLIN1 led to increased levels of UPR and autophagy-associated protein functions (Figure 3A–H). Next, we performed rescue experiments to validate whether ALKBH5-dependent regulation of the UPR occurs via ERLIN1. ERLIN1 overexpression rescued the induction of UPR proteins and autophagy in ALKBH5-silenced cells (Figure 3I–J).

ER Ca^2+^ homeostasis is critical for proper ER functioning, as Ca^2+^ depletion in the ER leads to ER stress and the induction of the UPR [23]. Since ERLIN1-dependent regulation of IP3R is an important regulator of Ca^2+^ flux, we asked whether ALKBH5 may be an important, yet hitherto, unrecognized regulator of IP3R-dependent Ca^2+^ flux in ER. Indeed, ALKBH5 silencing resulted in increased levels of IP3R (Figure 3K). Expectedly, ERLIN1 knockdown increased the level of IP3R (Figure 3L). In accordance with these results, either ALKBH5 or ERLIN1 efflux of Ca^2+^, as determined by flou8AM fluorescent staining (Figure 3K–O). To understand the impact of m^6^A-dependent regulation of intracellular calcium and its downstream effect on ER stress, we performed rescue experiments. ERLIN1 overexpression reversed the accumulation of intracellular calcium in ALKBH5-silenced cells (Figure 3P). These data suggest that ALKBH5-ERLIN1 signaling may promote ERAD-mediated degradation of IP3R to maintain the high level of luminal Ca^2+^ needed for the proper functioning of the ER.

Ca^2+^ flux between mitochondria and ER is inextricably linked with ER stress and mitochondrial function. For example, impaired Ca^2+^ influx can induce serine/threonine kinase AMP-activated protein kinase (AMPK), which regulates several aspects of mitochondrial function, including mitochondrial biogenesis and mitophagy. Moreover, AMPK regulates the transcription and activity of several genes involved in mitochondrial biogenesis and autophagy [24,25].

**Figure 4:**
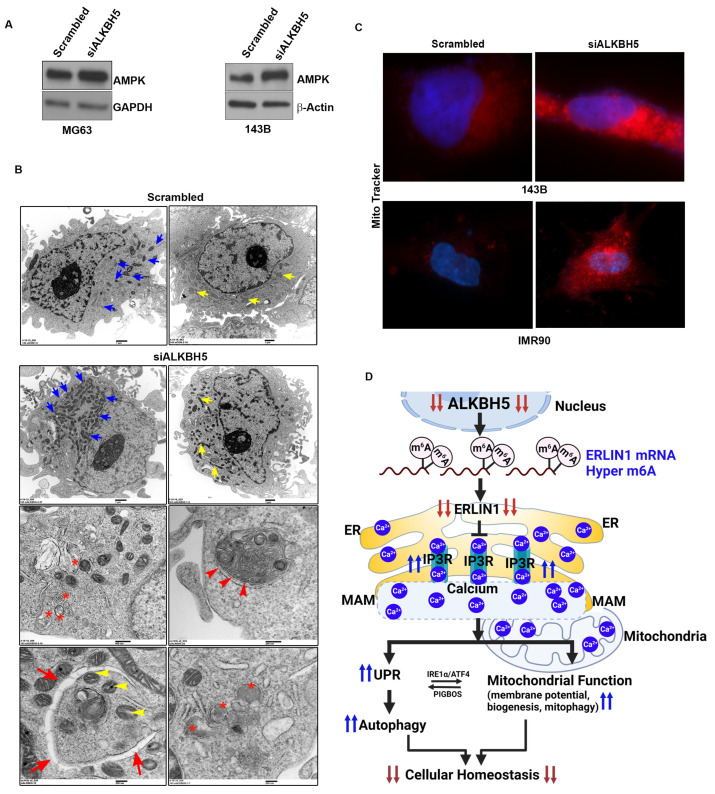
(**A**) Western blot analyses of scrambled-siRNA and siALKBH5 transfected cells using an antibody against AMPK. β-actin was used as a loading control. Gel photographs are representative of at least three independent experiments. (**B**) Transmission electron microscopy of 143B cells transfected with scrambled-siRNA or siALKBH5. Ultrastructure of 143B cells shows the number of mitochondria (blue arrow); yellow arrow shows the length of the mitochondria, red asterisks show phagosomes; red arrowhead shows autophagolysosomes; red arrow shows double membrane autophagosomes wrapping up mitochondria (yellow arrowhead), and lysosomes in 143B cells. (**C**) Immunofluorescence images showing the mitochondrial membrane potential of scramble/siALKBH5 transfected cells (top row, 143B cells; bottom row, IMR90 cells) were live-stained with MitoTracker dye for 30 min and observed under a fluorescence microscope. (**D**) Model showing how ALKBH5 may regulate cellular/ER homeostasis. Based on our results, we propose that ALKBH5 silencing leads to reduced stability of ERLIN1, which in turn blocks ERAD-mediated degradation of IP3R, which increases levels of IP3R. Increased IP3R leads to Ca^2+^ efflux from the ER lumen, resulting in ER stress. The Ca^2+^ efflux from the ER, leading to ER stress, induces the UPR and autophagy. The UPR, in turn, regulates the membrane (MAM)-mediated transfer of Ca^2+^ into the mitochondria. Several lines of evidence support a direct role for UPR proteins IRE-α and ATF4 in regulating MAM-mediated calcium signaling and mitochondrial function. UPR and mitochondrial crosstalk is also supported by studies showing induction of the UPR by mitochondria membrane-associated microprotein PIGBOS [26]. The transfer of Ca^2+^ from the ER to mitochondria changes the mitochondrial membrane potential and promotes mitochondrial biogenesis and mitophagy to meet the increased energy demands resulting from ER stress.

To test whether ALKBH5-IP3R-ERLIN1-dependent calcium flux may affect AMPK activity and mitochondrial function, we first determined AMPK levels in ALKBH5-silenced cells. ALKBH5 silencing induced the level of AMPK (Figure 4A). Next, we performed transmission electron microscopy (TEM) on ALKBH5-silenced cells. ALKBH5-depleted cells had significantly increased mitochondrial biogenesis compared to scrambled transfected cells (Figure 4B). Furthermore, ALKBH5 knockdown cells showed increased silencing and increased the levels of calcium-binding proteins calnexin and calreticulin (CALR) as well as the double membrane autophagy vesicles. To further confirm the impaired mitochondrial function in ALKBH5- silenced cells, we performed immunofluorescence analysis using MitoTracker™ dye (Thermo Fisher Scientific, Waltham, MA, USA), which stains mitochondria in live cells; its accumulation reflects changes in membrane potential. The fluorescent signal was increased in ALKBH5-depleted cells compared to scrambled-transfected cells, further supporting our finding that ALKBH5-IP3R-ERLIN1-dependent maintenance of Ca^2+^ levels regulates mitochondrial functions (Figure 4C).

## 4. Conclusions

In summary, we show that ALKBH5 is important in regulating ERLIN1-IP3R-dependent calcium flux between ER and mitochondria, and consequently, AMPK activation and mitochondrial biogenesis in both normal and cancer cells. These findings provide the other hand, sustained activation of the UPR (and therefore, activation of apoptosis signaling) can be exploited for therapeutic purposes such as treating cancer. Although the UPR is a critical pro-survival mechanism, its sustained activation may also induce apoptotic signaling; therefore, UPR activity must be tightly regulated for maintaining normal cellular homeostasis (Figure 4D). On the other hand, sustained activation of UPR (and therefore activation of apoptosis signaling) can be exploited for therapeutic purposes such as treating cancer. Our results showing the induction of the UPR in ALKBH5-silenced cancer cells suggest that inhibiting ALKBH5 may be a novel approach for new drugs to treat cancers that depend on ALKBH5 pro-tumorigenic signaling. However, our results also demonstrated that silencing of ALKBH5 altered calcium signaling and induced the UPR in normal cells.

Since ALKBH5 knockout mice are viable and anatomically normal, except for impaired fertility, our results suggest that normal cells and cancer cells may have different mechanisms and thresholds to negotiate ER stress. In support of that idea, calcium-signaling dynamics, such as the nature of Ca^2+^ influx and rate of Ca^2+^ recovery, differ in normal and cancer cells [27,28]. For example, treatment with thapsigargin, a SERCA inhibitor, slowed the return to baseline Ca^2+^ levels in cancer cells compared to normal cells [28]. It is also plausible that, unlike normal cells, cancer cells may be more addicted to ALKBH5, meaning they are more sensitive to changes in the levels of ALKBH5, and consequently, to the extent of the UPR activation.

## Data Availability

All data associated with this manuscript will be available upon request.

## Supplementary Materials

The following supporting information can be downloaded at: https://www.mdpi.com/article/10.3390/cells12091283/s1, Figure S1: Supplementary Figure S1; Table S1: Key resoruces.
