# Supplementary material for: The RNA Demethylase ALKBH5 Maintains Endoplasmic Reticulum Homeostasis by Regulating UPR, Autophagy, and Mitochondrial Function
Source: Cells. 2023 Apr 29;12(9):1283. doi: 10.3390/cells12091283 (PMC10177234; doi:10.3390/cells12091283)
Supplement: Supplementary file 1 [file cells-12-01283-s001.zip › cells-2320576-supplementary.pdf]

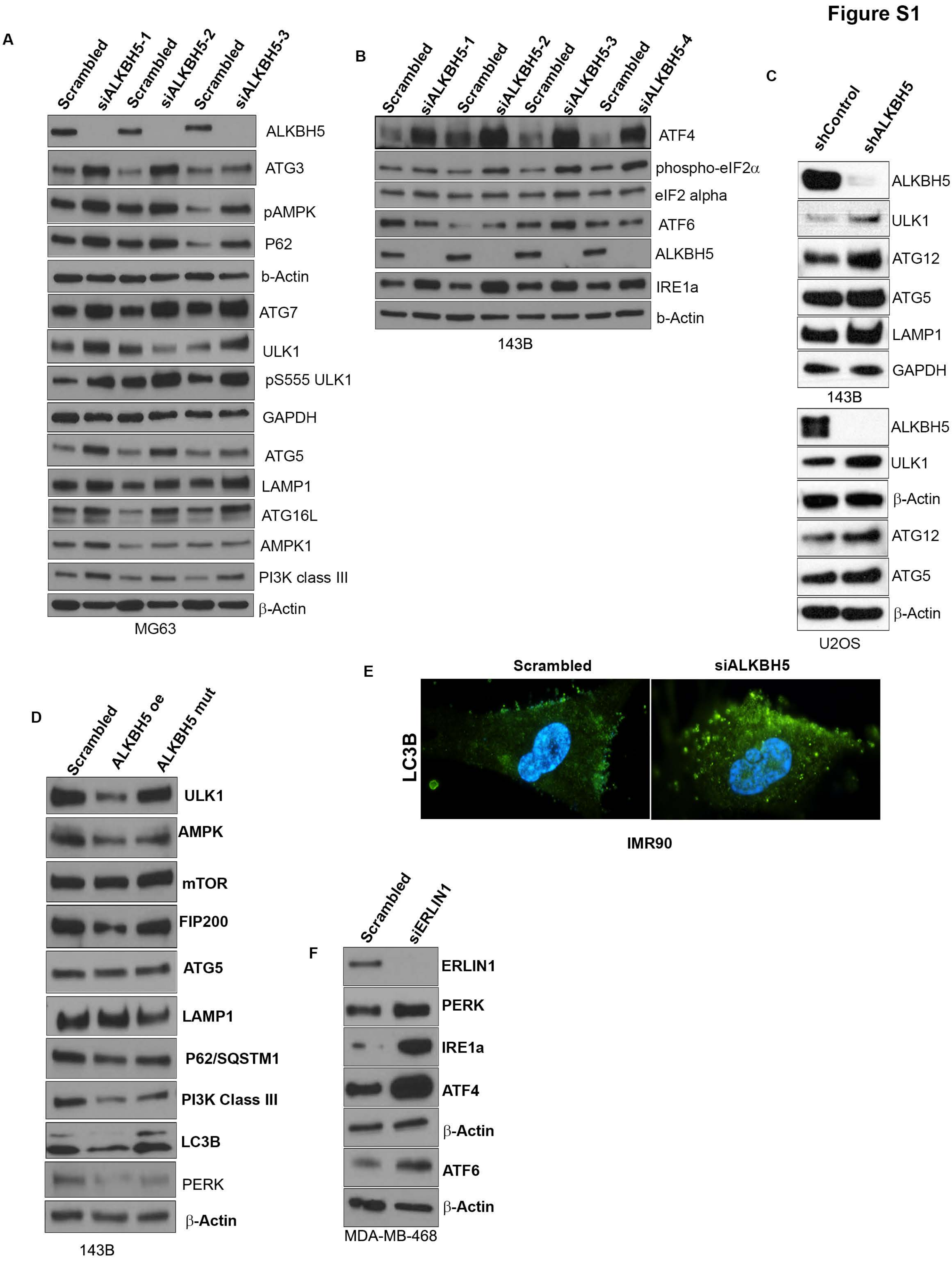

Figure S1
A
Scrambled
siALKBH5-1
Scrambled
siALKBH5-2
Scrambled
siALKBH5-3
ALKBH5
ATG3
pAMPK
P62
b-Actin
ATG7
ULK1
pS555 ULK1
GAPDH
ATG5
LAMP1
ATG16L
AMPK1
PI3K class III
β-Actin
MG63
B
Scrambled
siALKBH5-1
Scrambled
siALKBH5-2
Scrambled
siALKBH5-3
Scrambled
siALKBH5-4
ATF4
phospho-eIF2α
eIF2 alpha
ATF6
ALKBH5
IRE1a
b-Actin
143B
C
shControl
shALKBH5
ALKBH5
ULK1
ATG12
ATG5
LAMP1
GAPDH
143B
ALKBH5
ULK1
β-Actin
ATG12
ATG5
β-Actin
U2OS
D
Scrambled
ALKBH5 oe
ALKBH5 mut
ULK1
AMPK
mTOR
FIP200
ATG5
LAMP1
P62/SQSTM1
PI3K Class III
LC3B
PERK
β-Actin
143B
E
Scrambled
siALKBH5
LC3B
IMR90
F
Scrambled
siERLIN1
ERLIN1
PERK
IRE1a
ATF4
β-Actin
ATF6
β-Actin
MDA-MB-468

Table S1: Key resources Table

Antibody	Source	Identifier
ALKBH5	Sigma	HPA007196
METTL3	Sigma	Anti-METTL3 antibody produced in rabbit #SAB2104747
METTL14	Sigma	Anti-METTL14 antibody produced in rabbit HPA038002
FTO	Sigma	Anti-FTO antibody produced in rabbit HPA041086
ALKBH5	sigma	Anti-ALKBH5 antibody produced in mouse SAB1407587
FTO	Phosphosolutions	Anti-FTO (Fat Mass and Obesity Related Protein) Antibody (597-FTO)
ATG3	Cell Signaling Technologies	Atg3 Antibody #3415
ATG5	Cell Signaling Technologies	Atg5 (D5F5U) Rabbit mAb #12994
ATG7	Cell Signaling Technologies	Atg7 (D12B11) Rabbit mAb #8558
AMPK	Cell Signaling Technologies	AMPKα Antibody #2532
P62/SQTM	Sigma	Anti-P62 DOK antibody produced in rabbit
P62/SQTM	Cell Signaling Technologies	SQSTM1/p62 (D1Q5S) Rabbit mAb #39749
GAPDH	Santa Cruz Biotechnology	Anti-GAPDH Antibody (6C5): sc-32233
ULK1	Cell Signaling Technologies	ULK1 (D8H5) Rabbit mAb #8054
LAMP1	Cell Signaling Technologies	LAMP1 (D2D11) XP® Rabbit mAb #9091
LAMP1	Santa Cruz Biotechnology	Anti-LAMP-1 Antibody (H4A3): sc-20011
ATG16L	Cell Signaling Technologies	Atg16L1 (D6D5) Rabbit mAb #8089
PI3K classIII	Cell Signaling Technologies	PI3 Kinase Class III (D9A5) Rabbit mAb #4263
LC3B	Cell Signaling Technologies	LC3B Antibody #2775
LC3B	Sigma	Anti-LC3B antibody, Mouse monoclonal
Calrecticulin (CALR)	Cell Signaling Technologies	Calreticulin (D3E6) XP® Rabbit mAb #12238
Calnexin	Cell Signaling Technologies	Calnexin (C5C9) Rabbit mAb #2679
IP3R	Abcam	Anti-IP3 receptor antibody (ab5804)
ATF4	Cell Signaling Technologies	ATF-4 (D4B8) Rabbit mAb #11815
PERK1	Cell Signaling Technologies	PERK (C33E10) Rabbit mAb #3192
IRE1alpha	Cell Signaling Technologies	IRE1α (14C10) Rabbit mAb #3294
ATF6	Cell Signaling Technologies	ATF-6 (D4Z8V) Rabbit mAb #65880
ERLIN1	Santa Cruz Biotechnology	Anti-SPFH1 Antibody (A-7): sc-514820
eIF2alapha	Cell Signaling Technologies	eIF2α (D7D3) XP® Rabbit mAb #5324
phospho eIF2alpha	Cell Signaling Technologies	Phospho-eIF2α (Ser51) (D9G8) XP® Rabbit mAb #3398
m6A RNA IP kit	Sigma EMD Millipore	EpiQuik m6A RNA Methylation Quantification Kit (Colorimetric) P-9005-48

RNA IP kit	Sigma EMD Millipore	Magna RIP™ RNA-Binding Protein Immunoprecipitation Kit #17-700
global m6A measuring kit	Epigentek	EpiQuik m6A RNA Methylation Quantification Kit (Colorimetric)
Scrambled siRNA	Sigma	SIC001 Universal Negative Control#1
siALKBH5-1	Sigma	**SASI_Hs02_00350577**
siALKBH5-2	Sigma	**SASI_Hs01_00013942**
siALKBH5-3	Sigma	**SASI_Hs01_00013943**
siALKBH5-4	Sigma	SASI_Hs01_00013947
siALKBH5-5	Sigma	**SASI_Hs01_00013948**
siMETTL14	Sigma	SASI_Hs01_00179440
siMETTL3	Sigma	SASI_Hs01_00044317
siERLIN1	Sigma	SASI_Hs02_00322324
siFTO	Sigma	SASI_Hs02_00314786
mitotracker	Cell Signaling Technologies	MitoTracker® Deep Red FM #8778
ERLIN1 oe Plasmid	Genscript	Genscript Catalog No: OHu25510
siYTHDF1	Sigma	SASI_Hs01_00233686
siYTHDF2	Sigma	SASI_Hs01_00133214
siYTHDF3	Sigma	SASI_Hs01_00202277
siIGF2BP1	Sigma	SASI_Hs01_00074473
siIGF2BP2	Sigma	SASI_Hs01_00166607
siIGF2BP3	Sigma	SASI_Hs01_00207964
siALKBH5	Santa cruz	sc-93856
siALKBH5	Dharmacon	Catalog ID:L-004281-01-0005
Flou8AM	Abcam	Fluo-8AM green fluorescent calcium binding dye (ab142773)
